# Companion Diagnostic FDA Review Flexibilities: An Assessment of CDx for NSCLC to Support Aligned Approaches for Validation

**DOI:** 10.1007/s43441-025-00799-7

**Published:** 2025-05-15

**Authors:** Hillary S. Andrews, Grace Collins, Bernat Navarro-Serer, Mark D. Stewart, Jeff D. Allen

**Affiliations:** Friends of Cancer Research, 1800 M Street NW Suite 1050S, Washington, DC 20036 USA

## Abstract

The U.S. Food and Drug Administration (FDA) recommends concurrent development of targeted therapies with an associated companion diagnostic (CDx) as the optimal approach to provide patient access to novel, safe, and effective treatments. However, CDx validation often relies on clinical samples from pivotal clinical trials for the drug, which can be challenging, particularly when there is limited sample availability. A review of Summary of Safety and Effectiveness Data (SSED) documents for CDx approved for non-small cell lung cancer (NSCLC) revealed that CDx for rare biomarkers often use alternative samples for validation. While the practice of using alternative samples for validation occurs, it is not always clear when these flexibilities are considered or how alternative samples should be used for validation. To address this, we propose the FDA establish guidance for the use of alternative sample sources for CDx validation, especially for rare biomarkers, to ensure timely and effective patient access to targeted therapies.

## Background

Precision medicine is transforming cancer care by enabling the development of therapies tailored to specific biomarkers. However, the success of these therapies depends on reliable companion diagnostics (CDx) that can accurately match patients to treatments. For rare biomarkers, limited sample availability poses unique challenges to CDx validation, potentially delaying patient access to beneficial treatments.

In most instances, the U.S. Food and Drug Administration (FDA) recommends concurrent development of a CDx alongside drugs targeting a specific biomarker [1, 2]. However, this concurrent approval process does not always occur and targeted therapies have, in some cases, been approved without an accompanying CDx for patient selection, particularly when therapeutic clinical trials are expedited to address an unmet need or involve small patient populations [3]. Delayed CDx approval is not preferable, and the application of regulatory flexibility enabling the use of alternative sample sources for CDx validation is one approach that could help address these challenges. However, it requires a clear understanding of when and how such flexibilities should be applied [4]. 

CDx review and subsequent approval focus on analytical and clinical validation of the test, while assessing safety and effectiveness. For more prevalent biomarkers, FDA recommends clinical validation be performed using samples from the pivotal clinical trial that supports the drug’s approval. However, in some cases, FDA guidance acknowledges it may be infeasible to acquire a sufficient number of clinical samples from the pivotal study for retesting, necessitating alternative approaches to validation, such as using a subset of trial samples. Should alternative validation approaches be used, FDA outlines considerations for ensuring validation studies reflect test performance in the intended use population [2]. 

## Review of SSED Documents from CDx Based on Rarity

Understanding how diagnostic test developers address these challenges in obtaining sufficient clinical samples during the validation process is critical for informing best practices and future regulatory requirements. To explore this, we reviewed Summary of Safety and Effectiveness Data (SSED) documents from the Premarket Approval (PMA) application available on FDA’s website for CDx used for non-small cell lung cancer (NSCLC). Findings revealed that alternative sample sources were frequently used when samples from pivotal clinical trials were limited (Table 1). For each CDx, we focused on the Primary Clinical Study section in the SSED and searched for terms related to alternative samples. These alternative sample sources included archival specimens, retrospective samples, and commercially acquired specimens. Interestingly, alternative samples were more commonly used for the rarest biomarkers (3/3 PMAs, 100% for the rarest biomarkers vs. 4/10 PMAs, 40% for the least rare biomarkers).

**Table 1:** Overview of alternative samples used for assessment of clinical performance

Biomarker Group	PMAs	# PMAs Using Alternative Sample Sources with Descriptions	PMAs Using Clinical Trial Samples
**Rarest** *Prevalence 1–2%* ROS1, [5] BRAF V600E [6]	3	3/3 PMAs • 139 archival specimens [7] • 305 retrospective melanoma samples not obtained from clinical trial [8] • 117 negative commercially acquired samples [7]	2/3
**Rare** *Prevalence 3–13%* ALK, [9] KRAS G12C [10]	5	2/5 PMAs • 148 supplemental matched tissue and plasma samples from commercial vendors [11] • 303 patients from separate trial used to evaluate concordance between the two sample types [12]	5/5
**Least Rare** *Prevalence 24–60%* EGFR exon 19 deletions, EGFR exon 21 L858R alterations, EGFR exon 20 T790M alterations, [13] PD-L1 [14]	10	4/10 PMAs • 282 retrospective samples not obtained from a clinical trial [8] • 130(-) FFPE NSCLC archival specimens sourced from commercial vendors [7] • The NILE study provided supplemental samples to calculate NPA (*N* = 92)(15) • 35 patients for whom data were previously generated on Guardant360 LDT [15]	9/10

In some cases, pivotal study enrollment is based on one or more Clinical Trial Assays, which may include the candidate CDx test as well as local tests performed at individual trial sites. Bridging studies are then performed to evaluate the agreement between assays (e.g., the enrollment tests vs. the candidate CDx) and to link clinical data from the intended use population to the candidate CDx. This process is critical to ensure the CDx can reliably provide clinically actionable results compared to the local trial assays and supports the demonstration of its safety, effectiveness, and approval. Most CDx included in this analysis required a bridging study (16/18, 89%). We analyzed the number of samples in these bridging studies and found that those for the rarest biomarkers had fewer positive (median 67 [25–167]) or negative samples (median 119 [114–135]), while the least rare biomarkers included the greatest number of positive (median 182.5 [72–282]) or negative (median 150 [106–277]) samples (Table 2).

**Table 2:** Bridging studies with median valid positive and negative samples included by biomarker prevalence

Biomarker Group	# of PMAs with Bridging Results	Valid Positive Samples Included in Bridging (Median (range))	Valid Negative Samples Included in Bridging (Median (range))
**Rarest ** *Prevalence 1–2%* ROS1, BRAF V600E	3/3	67 (25–167)	119 (114–135)
**Rare** *Prevalence 3–13%* ALK, KRAS G12C	4/5	82 (75–179)	145 (75–754)
**Least Rare** *Prevalence 24–60* **%** EGFR exon 19 deletions, EGFR exon 21 L858R alterations, EGFR exon 20 T790M alterations, PD-L1	9/10	182.5 (72–282)	150 (108–277)
**All**	**16/18**	**136 (25–282)**	142 **(75–754)**

### Regulatory Flexibilities and Consistency in Validation Strategies

Based on findings in the SSEDs, it is clear that regulatory flexibilities are often applied in situations where sample availability is limited. However, the lack of explicit regulatory guidance on when and how these samples should be used may create uncertainty for developers. The FDA could draw on this prior experience to establish guidance clarifying situations in which regulatory flexibilities would be considered, as well as identifying the types of sample sources that could support robust validation and regulatory review.

Understanding when to use alternative sample sources could support sponsors in determining when to request flexibilities [16]. For instance, rare biomarkers, defined as a prevalence that is less than 1% of the overall population of patients with that specific cancer type, could serve as a starting point for considering flexibility. However, other factors limiting sample availability, along with considerations such as the technology or the intended use of the assay, may influence decisions to exercise flexibility. For example, blood-based biomarkers may warrant additional flexibility due to sample volume limitations.

Additionally, defining the types of data appropriate for different aspects of test validation could help sponsors select appropriate alternative data sources and prioritize limited clinical samples. For instance, cell lines such as immortalized cell lines or primary cultures, could be leveraged for analytical validation to assess interference, reagent stability, or guard banding. However, these would not be appropriate for clinical validation that requires outcomes data or other analytical studies that consider tissue complexity. For these types of studies, prioritization of clinical samples will be important. Overall, it is critical to consider which alternative sample type best represents the information that is necessary to garner from the validation analysis.

Sponsors seeking to use alternative sample sources should engage early with FDA through mechanisms such as pre-IDE meetings or Q-submissions. Early and clear communication with the FDA, supported by well-documented justifications, is critical when seeking flexibility for CDx validation to support an FDA approval. A consistent format for providing this information could streamline the sponsor discussions with FDA and the review process. For example, a document addressing validation study design, proposed samples, sample sources, and justifications for using the samples for each proposed validation study would allow reviewers to efficiently assess strategies [16]. 

## Conclusion

Regulatory flexibilities play a critical role in CDx development when limited samples are available from the pivotal clinical trial. The FDA has considerable experience supporting the use of alternative samples for clinical and analytical validation. Establishing formal guidance on these flexibilities would facilitate more streamlined processes and support sponsors in incorporating alternative samples into their validation strategies, as appropriate. The types of alternative samples appropriate for different validation purposes should be clearly defined to ensure robust validation while maintaining regulatory standards. Additionally, a consistent, well-documented approach across development programs could facilitate efficient communication between sponsors and FDA reviewers, ultimately ensuring that biomarker-based precision therapies reach patients more effectively.

## Data Availability

No datasets were generated or analyzed during the current study.
